# Circulating Adaptive Immune Cells Expressing the Gut Homing Marker α4β7 Integrin Are Decreased in COVID-19

**DOI:** 10.3389/fimmu.2021.639329

**Published:** 2021-04-20

**Authors:** Tanja M. Müller, Emily Becker, Maximilian Wiendl, Lisa Lou Schulze, Caroline Voskens, Simon Völkl, Andreas E. Kremer, Markus F. Neurath, Sebastian Zundler

**Affiliations:** ^1^ Department of Medicine 1 and Deutsches Zentrum Immuntherapie (DZI), University Hospital Erlangen, Friedrich-Alexander-Universität Erlangen-Nürnberg, Erlangen, Germany; ^2^ Department of Dermatology, University Hospital Erlangen, Friedrich-Alexander-Universität Erlangen-Nürnberg, Erlangen, Germany; ^3^ Department of Internal Medicine 5, Hematology and Clinical Oncology, University Hospital Erlangen, Friedrich-Alexander-Universität Erlangen-Nürnberg, Erlangen, Germany

**Keywords:** COVID-19, SARS-CoV-2 infection, T cell trafficking, integrins, gut homing

## Abstract

**Background:**

Infection with the novel severe acute respiratory syndrome coronavirus 2 (SARS-CoV-2) causes a wide range of symptoms including gastrointestinal manifestations, and intestinal epithelial cells are a target of the virus. However, it is unknown how the intestinal immune system contributes to systemic immune responses in coronavirus disease 2019 (COVID-19).

**Methods:**

We characterized peripheral blood lymphocytes from patients with active COVID-19 and convalescent patients as well as healthy controls by flow cytometry.

**Results:**

The frequency and absolute number of circulating memory T and B cells expressing the gut homing integrin α4β7 integrin was reduced during COVID-19, whether gastrointestinal symptoms were present or not. While total IgA-expressing B cells were increased, gut-imprinted B cells with IgA expression were stable.

**Conclusion:**

COVID-19 is associated with a decrease in circulating adaptive immune cells expressing the key gut homing marker α4β7 suggesting that these cells are preferentially recruited to extra-intestinal tissues independently of α4β7 or that the systemic immune response against SARS-CoV-2 is at least numerically dominated by extraintestinal, particularly pulmonary, immune cell priming.

## Introduction

The emergence of SARS-CoV-2 in late 2019 (1) has led to a global pandemic that is still far from being under control in many parts of the world. The disease caused by SARS-CoV-2 has been termed COVID-19, which is typically characterized by pneumonia potentially manifesting as fatal respiratory distress syndrome (2). However, it has already early been described that a subgroup of patients with COVID-19 also shows gastrointestinal symptoms like diarrhea or vomiting (2–6) and SARS-CoV-2 RNA is frequently detected in the feces of COVID-19 patients (7). Consistently, productive infection of enterocytes in the human gut has been demonstrated (8). Mechanistically, this observation could be linked to the expression of angiotensin-converting enzyme 2 (ACE2), which serves as the entry receptor for the spike protein of SARS-CoV-2 (9), not only in the lung, but also in the digestive tract (10, 11).

As the organ with the largest surface of the body, the gastrointestinal tract faces the challenge to allow absorption of nutrients, vitamins and minerals, while protecting against potentially harmful luminal microbiota or toxic products (12). Thus, a complex immune system has established in the gut including the secondary lymphoid organs of the gut-associated lymphoid tissue (GALT) as well as immune cells in the lamina propria of the intestinal mucosa (13). A hallmark and exclusive feature of immune cell priming in the GALT is that dendritic cells (DCs) expressing the enzyme retinaldehyde dehydrogenase (RALDH) metabolize nutritional vitamin A to retinoic acid. This induces the expression of the gut homing integrin α4β7 and the chemokine receptor CCR9 on T cells receiving antigenic stimulation from these DCs (14–16) and offers the opportunity to specifically track lymphocytes imprinted in the intestine. Moreover, GPR15 has been described to be specifically expressed on T lymphocytes homing to the large intestine (17, 18).

The α4β7 integrin specifically binds to mucosal vascular addressin cell adhesion molecule (MAdCAM)-1 (19, 20), which is virtually exclusively expressed on high endothelial venules in the gastrointestinal tract (21). Hence, upon recirculation, memory T cells primed within the GALT dispose of a particular signature to re-enter intestinal tissue or GALT. Moreover, retinoic acid generated by intestinal DCs has been shown to promote IgA-secreting B cells, at least partly by inducing an isotype switch towards IgA (22, 23). Thus, intestinal infection with SARS-CoV-2 might lead to the induction of virus-specific T cells with a gut-homing signature and IgA-producing B cells in the intestine. Importantly, IgA is present on surfaces and participates in upholding mucosal immunity. IgA produced by B cells shaped in the gut might therefore also lead to cross-protection of the mucosal surface of the respiratory tract and, consistently, oral vaccination strategies against SARS-CoV-2 have been advocated and are under development (24).

We therefore set out to investigate, whether infection with SARS-CoV-2 leads to systemic signs of virus-associated intestinal T and B cell immunity in the circulation. We show that in patients with COVID-19 α4β7-expressing memory T cells are reduced compared with healthy controls even in patients with intestinal symptoms, while IgA-producing B cells are stable. However, these do not seem to originate from the gut, suggesting that gut-imprinted immune cells are eliminated from the circulation or that immune responses generated at other sites of virus entry are dominating circulating immune cell profiles in COVID-19.

## Methods

### Patient Cohort

Blood for PBMC isolation was collected following informed written consent at the University Hospital Erlangen. Hospitalized patients with active SARS-CoV-2 infection (n = 110), recovered patients presenting to the Department of Transfusion Medicine (n = 35) and healthy donors (n = 28) were included.

Clinical data of COVID-19 patients were retrieved from an internal database. Clinical data of COVID-19 patients and healthy donors are summarized in **Table 1**, clinical data for the recovered patients are not available. Blood collection was approved by the Ethics Committee of the Friedrich-Alexander University Erlangen-Nuremberg (174_20B).

**Table 1:** Baseline characteristics of patients with SARS-CoV-2 infection and healthy controls.

		Healthy controls	COVID-19 patients
Number		28	110
Male [%]		28	64
Female [%]		72	36
Ø Age in years		28.8	63.5
Diarrhea		/	7
Course of disease	Mild	/	17
	Severe	/	17
Pre-existing cardiovascular disease		/	17

### PBMC Isolation and Flow Cytometry

Human peripheral blood mononuclear cells (PBMCs) were isolated by standard density gradient centrifugation with Ficoll Paque (GE) or Lymphocyte Separation Medium (Anprotec) and stained with the following antibodies:

CD3-APC (HIT3a, BioLegend), CD3-BV605™ (OKT3, BioLegend), CD4-APC-Vio770 (VIT4, Miltenyi Biotec), CD4-PerCP/Cy5.5 (OKT4, BioLegend), CD8a-PerCP/Cy5.5 (RPA-T8, BioLegend), CD19-VioBlue (LT19, Miltenyi Biotec), CD45RO-BV510™ (UCHL1, BioLegend), CD69-PE/DAZZLE™ 594 (FN50; BioLegend), CD154-FITC (24-31, BioLegend), CCR9-PE/Cy7 (L053E8, BioLegend) Integrin α4-PE/Cy7 (9F10, BioLegend), Integrin α4-VioBlue (MZ18-24A9, Miltenyi Biotec) Integrin β1-PE (TS2/16, BioLegend), Integrin β7-BV605^TM^ (FIB504, BioLegend), GPR15-PE (SA302A10, BioLegend), IgA-FITC (ab97219, abcam), Vedolizumab (Takeda Pharmaceuticals) labeled with Alexa Fluor^®^ 488 (Invitrogen) or Alexa Fluor^®^ 647 (Invitrogen).

PBMCs were washed for 5 min in PBS at 300x g and 4°C and fixed over night at 4°C using the Foxp3 transcription buffer staining kit (Thermo Fisher). Subsequently, PBMCs were washed, resuspended in 200µl FACS buffer (1% FBS, 2mM EDTA in PBS) and analyzed on a MacsQuant16 instrument (Miltenyi).

In some experiments, full blood was stained for flow cytometry as follows: 200µl peripheral whole blood was incubated for 15 minutes with 2ml Lysing Buffer (BD Pharm Lyse™, BD Biosciences) to lyse red blood cells. Samples were washed and subjected to antibody staining as described above. After fixation and washing of the samples exactly 200µl FACS buffer was added and the samples were analyzed on a MacsQuant16 instrument.

### SARS-CoV-2 Specific T Cell Stimulation

SARS-CoV-2-specific T cell response was analyzed by stimulating PBMCs from COVID-19 patients and healthy controls with a pool of peptides covering the immunodominant sequence domains of the surface glycoprotein of SARS-CoV-2 (PepTivator^®^ SARS-CoV-2 Prot_S-research grade, Miltenyi Biotec) for 5 hours according to the manufactures instructions. Subsequently, stimulated PBMCs were stained with antibodies as described above and analyzed on a MacsQuant16 instrument. SARS-CoV-2-specific T cells were detected with antibodies against CD69 and CD154.

### Statistical Analysis

GraphPad Prism (GraphPad Software, Inc.) was used to perform statistical analyses.

Results are shown as scatter dot plots with individual data points. Center values and error bars represent mean and standard error of the mean (SEM).

Normal distribution was tested using Shapiro-Wilk test. When comparing two groups, statistical differences were tested using student’s T test for data with normal distribution or Mann Whitney U test for data without a normal distribution. When comparing more than two groups, statistical differences were tested using one-way ANOVA with Tukey’s multiple comparison for data with a normal distribution or Kruskal–Wallis test with Dunn’s multiple comparison for data without a normal distribution. For correlation analyses, Spearman correlation was performed and a regression line is indicated. An α value of p < 0.05 was defined as statistically significant.

Significance levels are indicated by asterisks (* p < 0.05, ** p < 0.01, *** p < 0.001).

## Results

### α4β7 Integrin-Expressing Memory T Cells Are Decreased in COVID-19

We characterized T and B cells in the peripheral blood from a cohort of 80 patients with active COVID-19 and 35 patients recovered from COVID-19 as well as 18 healthy controls by flow cytometry of PBMCs. Clinical information on the patients is provided in **Table 1**. The overall frequency of CD3^+^ and CD3^+^CD4^+^ T cells was similar in patients with active or previous COVID-19 and in controls (**Supplementary Figures 1A, B**). Peripheral CD45RO^+^ memory CD4^+^ T cell and CD3^-^CD19^+^ B cell frequencies in patients with active or after COVID-19 were numerically, but not significantly increased compared with healthy donors (**Supplementary Figures 1C, D**).

**Figure 1:**
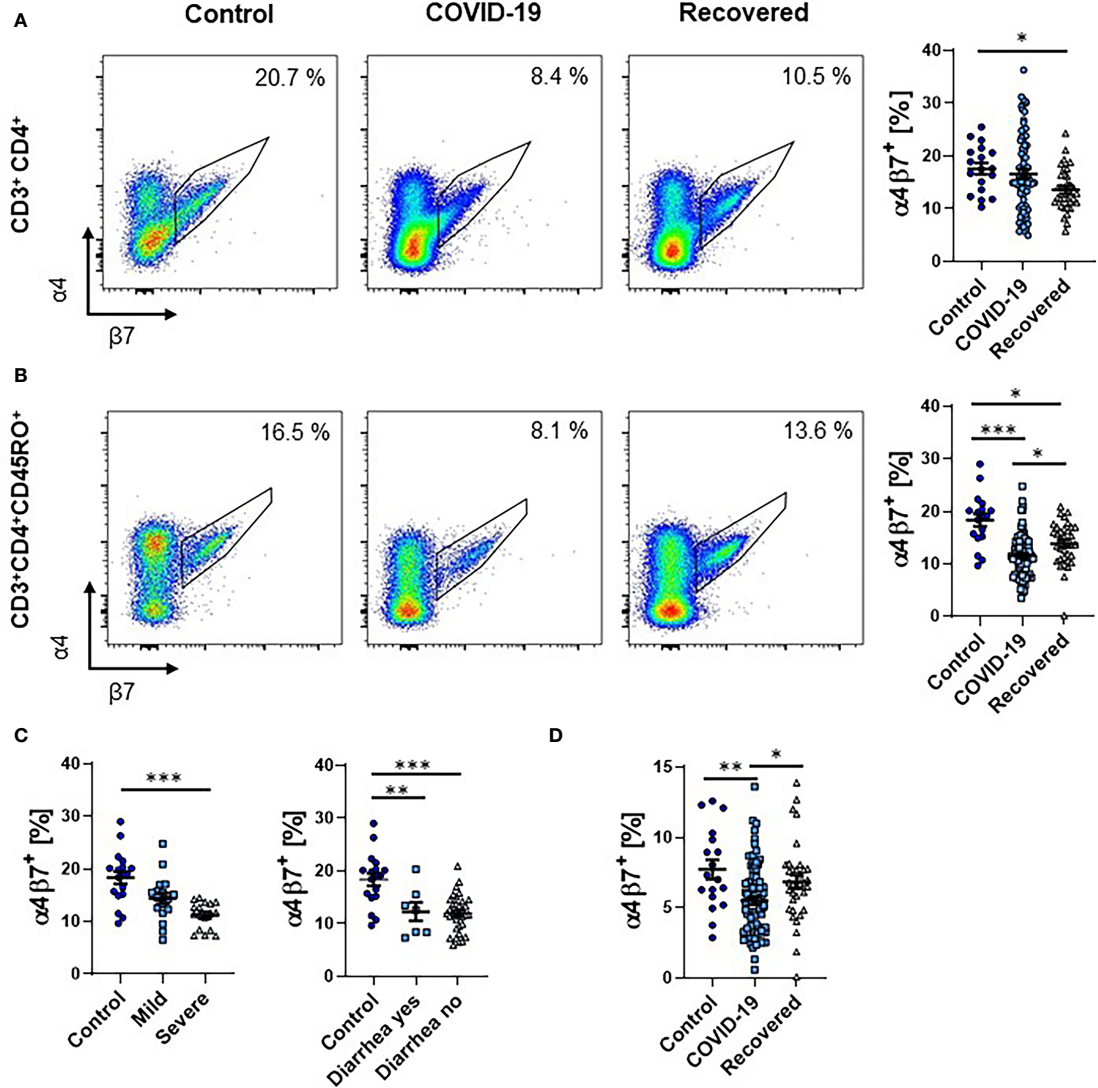
Frequency of α4^+^β7^+^ T cells during COVID-19. **(A, B)** Representative (left) and quantitative (right) flow cytometry of the frequency of α4β7 integrin-expressing CD3^+^CD4^+^ T cells **(A)** and CD3^+^CD4^+^CD45RO^+^ memory T cells **(B)**. **(C)** Quantitative flow cytometry of the frequency of α4β7 integrin-expressing CD3^+^CD4^+^CD45RO^+^ memory T cells in COVID-19 patients with mild or severe disease course (left) and with or without diarrhea (right) compared to healthy controls. **(D)** Quantitative flow cytometry of the frequency of α4β7 integrin-expressing CD3^+^CD4^+^CD45RO^+^ memory T cells expressed as frequency of CD3^+^CD4^+^ T cells. Each symbol represents an individual subject, n = 7 - 80 per group. *p < 0.05, **p < 0.01, ***p < 0.001.

To explore the presence of T cells primed in the intestine during SARS-CoV-2 infection, we analyzed the expression of α4β7 integrin on T cells in the peripheral blood. Interestingly, the portion of CD3^+^CD4^+^ T cells expressing α4β7 was substantially reduced in previous COVID-19 patients compared to healthy controls (**Figure 1A**). On CD3^+^CD4^+^CD45RO^+^ memory T cells, we observed a clear reduction of α4β7 expression in patients with active COVID-19. However, expression increased in the recovery phase, while still being lower than in controls (**Figure 1B**). Interestingly, there was no difference between patients with mild and severe course of COVID-19 as well as between patients with and without diarrhea (**Figure 1C** and **Supplementary Figure 2**). Even when relating the expression of α4β7 on CD45RO^+^ memory T cells to total CD3^+^CD4^+^ T cells, frequencies were decreased in COVID-19 patients compared to healthy controls (**Figure 1D**).

**Figure 2:**
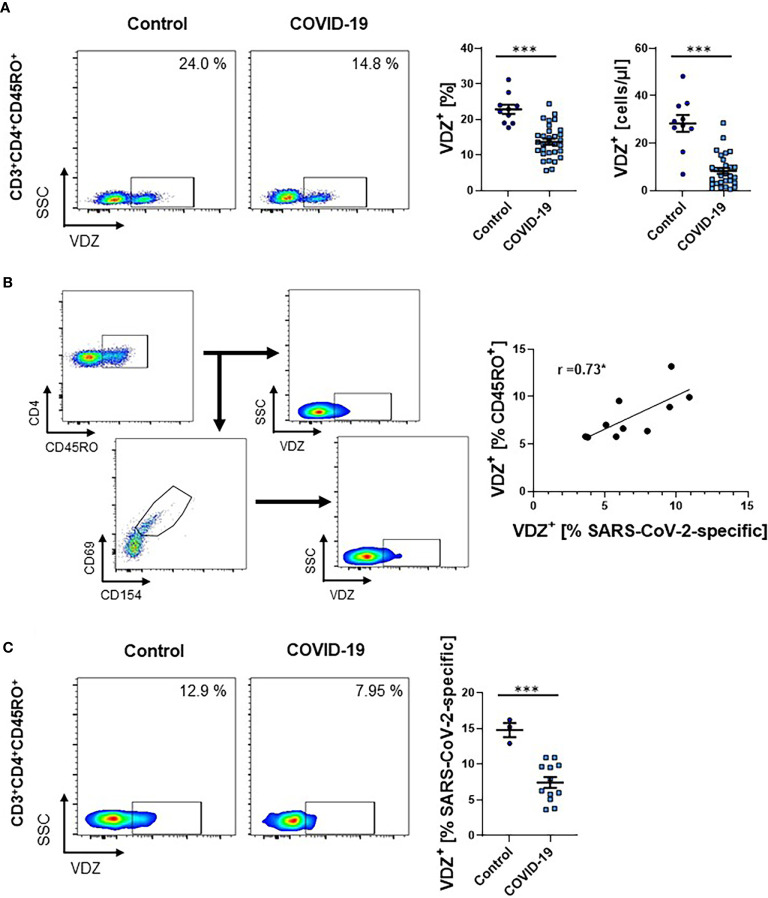
Frequency of VDZ^+^ CD4^+^ memory T cells during COVID-19. **(A)** Representative (left) and quantitative (right) flow cytometry of whole blood samples of COVID-19 patients and healthy controls. Graphs indicate the frequency and absolute cell numbers of CD3^+^CD4^+^CD45RO^+^ memory T cells staining positive for the anti-α4β7 integrin antibody VDZ. n = 10-31. **(B)** Exemplary gating strategy (left) and correlation (right) of α4β7 (VDZ) expression on SARS-CoV-2-specific CD3^+^CD4^+^CD45RO^+^ memory T cells to α4β7 (VDZ) expression on overall CD3^+^CD4^+^CD45RO^+^ memory T cells from patients with COVID-19. Spearman’s r is indicated. n = 10. **(C)** Representative (left) and quantitative (right) flow cytometry of the frequency of α4β7 (VDZ) expression on SARS-CoV-2-specific CD3^+^CD4^+^CD45RO^+^ memory T cells of COVID-19 patients and healthy controls. VDZ, vedolizumab. Each symbol represents an individual subject, n = 3 -12 per group. ***p < 0.001.

Since these data were only indicating relative expression and not absolute cell numbers, and did not exclude the possibility of co-expression of α4 and β7 integrin without heterodimerization, we seeked to validate our findings in an additional patient cohort. Here, we stained full blood samples and used fluorescently labeled vedolizumab, a monoclonal antibody specific for the α4β7 heterodimer used for the therapy of inflammatory bowel diseases (25). In accordance with previous literature marked lymphopenia was present in COVID-19 patients (**Supplementary Figure 3**). Corroborating our previous observations, the fraction as well as the absolute number of CD3^+^CD4^+^CD45RO^+^ T cells staining positive for vedolizumab was markedly reduced in patients with COVID-19 (**Figure 2A**).

To estimate, in how far expression of α4β7 integrin on overall memory CD4^+^ T cells reflects α4β7 expression of SARS-CoV-2-induced memory T cells, we used a cocktail of viral epitopes to stimulate PBMCs from COVID-19 patients. Flow cytometry demonstrated that there was a high degree of correlation of α4β7 expression between these cell subsets (**Figure 2B**). Moreover, we compared the expression of α4β7 on SARS-CoV-2-specific CD4^+^ memory T cells from COVID-19 patients with healthy donors without SARS-CoV-2 infection that also had SARS-CoV-2-specific T cells in their peripheral blood as previously reported (26). Again, we observed a striking decrease in the expression of α4β7 on these cells in COVID-19 patients compared with controls (**Figure 2C**), suggesting that circulating T cells primed in the gut are reduced in hospitalized patients with acute SARS-CoV-2 infection.

### GPR15, CCR9 and α4β1 Are Not Specifically Reduced in COVID-19

**Figure 3:**
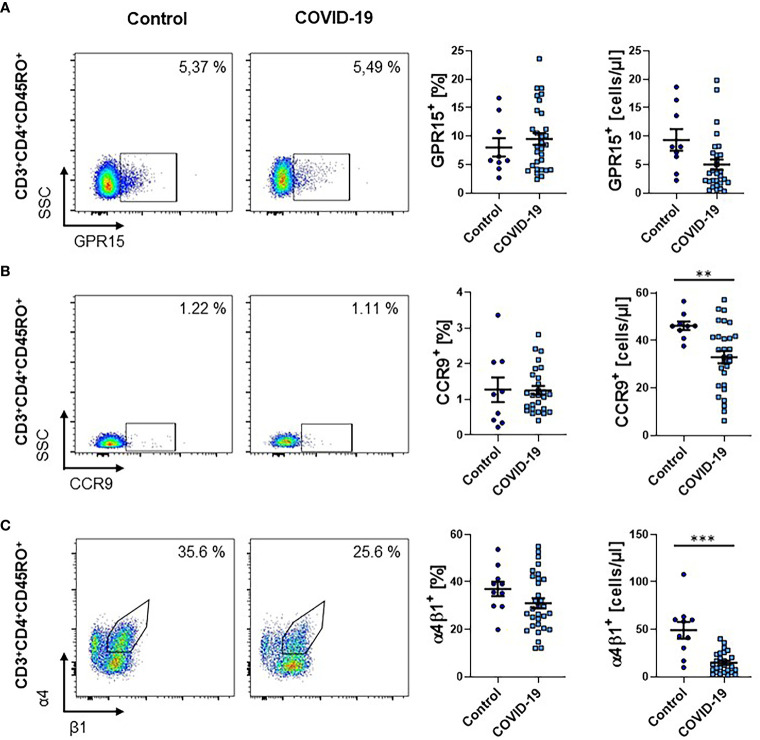
Frequency of other gut homing markers on CD4^+^ memory T cells during COVID-19. **(A–C)** Flow cytometry of whole blood samples from COVID-19 patients and healthy controls. Representative (left) and quantitative (right) flow cytometry of the frequency and absolute cell numbers of GPR15^+^ **(A)**, CCR9^+^ **(B)** and α4^+^β1^+^ **(C)** CD3^+^CD4^+^CD45RO^+^ memory T cells. VDZ, vedolizumab. Each symbol represents an individual subject, n = 10 – 31 per group. **p < 0.01, ***p < 0.001.

Next, we aimed to determine, whether T cells expressing gut-homing chemokine receptors are similarly reduced. Accordingly, we quantified the expression of GPR15, a receptor specifically expressed on T cells homing to the large intestine (17, 18), in full blood samples by flow cytometry. We observed no significant change of GPR15 expression in patients with active or after COVID-19 on CD4^+^ memory T cells (**Figure 3A**).

While the fraction of CD4^+^ memory T cells expressing the chemokine receptor CCR9, which is associated with trafficking to the small intestine (27), was similar between patients with COVID-19 and healthy controls, their absolute numbers in the circulation were significantly reduced (**Figure 3B**). We therefore decided to further investigate the abundance of memory T cells expressing the integrin α4β1, which has been implicated in homing to the small intestine (28). We observed a trend towards a reduction in the frequency of CD4^+^ memory T cells expressing α4β1 and a significant reduction in the absolute number of these cells in the circulation (**Figure 3C**).

Together, these effects were largely driven by the lymphopenia present in COVID-19 patients and, thus, the data suggested that infection with SARS-CoV-2 does not affect the expression of other gut-homing markers to a similar extent as α4β7.

### Reduction of Circulating α4β7-Expressing CD8^+^ T Cells in COVID-19

**Figure 4:**
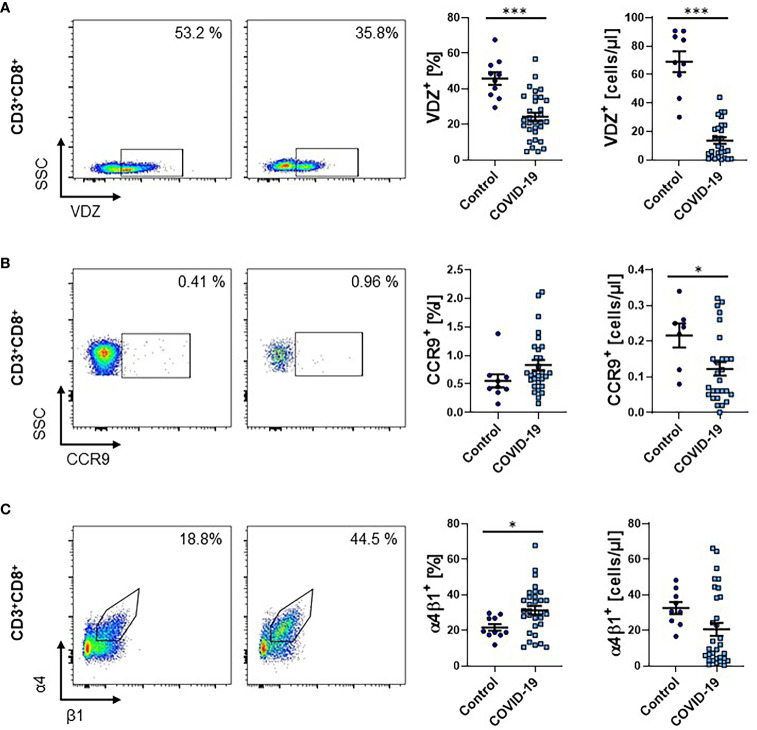
Frequency of gut homing markers on CD3^+^CD8^+^ T cells during COVID-19. **(A–C)** Flow cytometry of whole blood samples from COVID-19 patients and healthy controls. Representative (left) and quantitative (right) flow cytometry of the frequency and absolute cell numbers of α4β7 integrin- **(A)**, CCR9- **(B)** and α4β1 integrin- **(C)** expressing CD3^+^CD8^+^ T cells. VDZ, vedolizumab. Each symbol represents an individual subject, n = 10 – 31 per group. *p < 0.05, ***p < 0.001.

Since CD8^+^ T cells are crucially implicated in the immune response against viruses, we also explored the expression of gut-homing markers on CD8^+^ T cells during COVID-19. The percentage as well as the absolute number of CD3^+^CD8^+^ T cells in the peripheral blood of patients with COVID-19 was clearly reduced (**Supplementary Figure 4**). As in the CD4 compartment, a smaller fraction of CD8^+^ T cells from COVID-19 patients expressed α4β7 integrin (as indicated by positive staining for vedolizumab) compared to healthy controls. Similarly, the absolute number of these cells was substantially reduced (**Figure 4A**). For CCR9, the relative expression was not significantly altered, but the absolute number of CCR9-expressing circulating CD8^+^ T cells was reduced (**Figure 4B**). This was similar for α4β1, although, here, the increase in relative expression was significant, while the decrease in absolute cell numbers was not (**Figure 4C**).

Collectively, these observations largely recapitulated our findings for CD4^+^ T cells.

### IgA-Producing B Cells Without a Gut-Homing Phenotype Are Increased in Response to SARS-CoV-2

**Figure 5:**
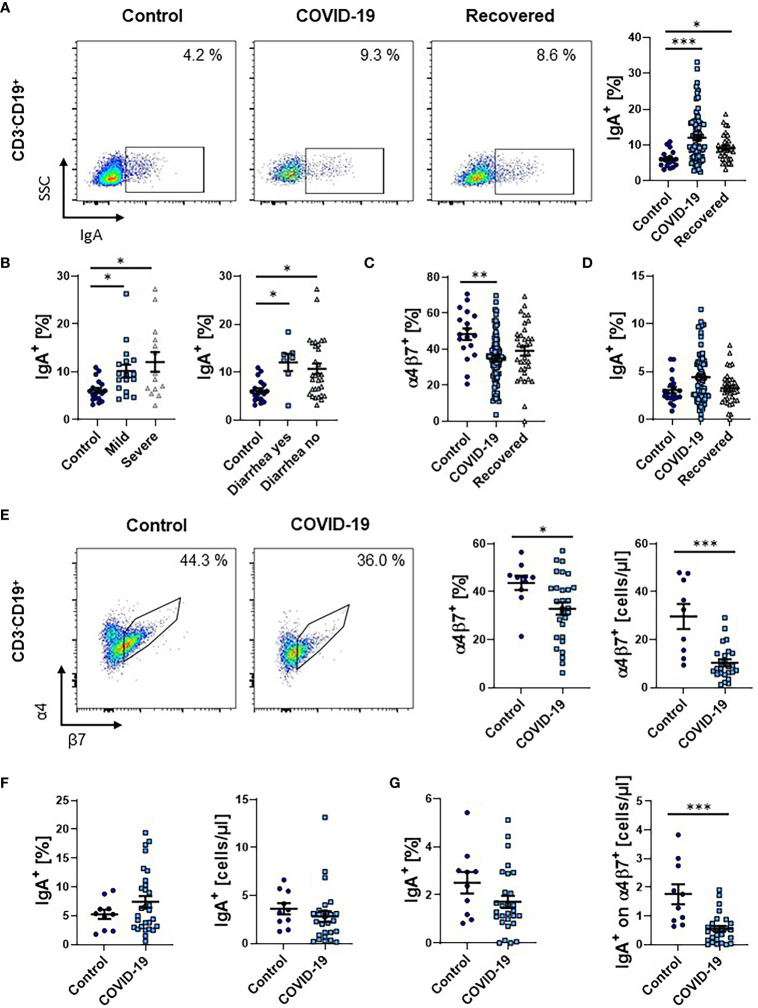
Frequency of IgA^+^ B cells during COVID-19. **(A)** Representative (left) and quantitative (right) flow cytometry of the frequency of IgA expressing CD3^-^CD19^+^ B cells from PBMCs. **(B)** Quantitative flow cytometry of the frequency of IgA-expressing CD3^-^CD19^+^ B cells in COVID-19 patients with mild or severe disease course (left) and with or without diarrhea (right) compared to healthy controls. **(C)** Quantitative flow cytometry of the frequency of α4β7 integrin-expressing CD3^-^CD19^+^ B cells. **(D)** Quantitative flow cytometry of the frequency of IgA^+^ cells among CD3^-^CD19^+^α4^+^β7^+^ B cells expressed as frequency of CD3^-^CD19^+^ B cells. **(E–G)** Flow cytometry of whole blood samples from COVID-19 patients and healthy controls. **(E)** Representative (left) and quantitative (right) flow cytometry of the frequency and absolute cell numbers of α4β7 integrin-expressing CD3^-^CD19^+^ B cells. **(F)** Quantitative flow cytometry of the frequency (left) and absolute cell numbers (right) of IgA-expressing CD3^-^CD19^+^ B cells. **(G)** Quantitative flow cytometry of the frequency (left) and absolute cell numbers (right) of IgA^+^ cells among CD3^-^CD19^+^α4^+^β7^+^ B cells expressed as frequency of CD3^-^CD19^+^ B cells. Each symbol represents an individual subject, n = 7 - 80 per group. *p < 0.05, **p < 0.01, ***p < 0.001.

Finally, we quantified the frequency of circulating CD19^+^ B cells expressing IgA. We observed a clear increase in patients with COVID-19 that persisted in the recovery phase (**Figure 5A**). This was more pronounced in patients with severe disease course, but similar in patients with and without gastrointestinal symptoms (**Figure 5B**).

Since a gut-homing phenotype including the expression of α4β7 integrin is also induced in B cells in the gut (23), we subsequently assessed the expression of α4β7 on these B cells. Similar to T cells, the frequency of α4β7-expressing B cells in patients with COVID-19 decreased and slightly re-increased in the recovery phase (**Figure 5C**). Interestingly, the increase in IgA-expressing B cells was not significant in the α4^+^β7^+^ subset (**Figure 5D**), supporting the notion that circulating IgA-expressing B cells during SARS-CoV-2 infection do not predominantly originate from the gut.

Again, we tried to reproduce our findings in a second patient cohort using full blood and staining with vedolizumab to detect α4β7 integrin. Similar to the analysis of PBMCs, α4β7-expressing B cells were significantly lower both on relative and absolute level (**Figure 5E**). Moreover, we again observed a numeric increase in the fraction of IgA-expressing B cells during COVID-19. Yet, this was not the case on absolute levels (**Figure 5F**). Notably, the expression of IgA on α4β7^+^ B cells was also lower in COVID-19 (**Figure 5G**).

Taken together, also in the B cell compartment, our data indicated that immune responses originating from the intestine in COVID-19 leave rather minor traits in the circulation.

## Discussion

The immune system is critical in the pathogenesis of COVID-19 (29, 30). Mediators released by infected cells initially recruit innate immune cells to the site of infection. This is the prerequisite for the subsequent generation of adaptive immune responses by presentation of SARS-CoV-2 epitopes to naïve T and B cells in local lymph nodes and the ensuing recruitment of antigen-experienced T cells to the site of inflammation as well as the production of specific antibodies by B cells (30). These processes are considered important for the clearance of the infection and the resolution of inflammation, but insufficient control, to the contrary, is involved in the development of acute respiratory distress syndrome and hyperinflammation in patients with severe disease course (31). Consistently, dexamethasone is an effective treatment in patients with severe COVID-19 (32).

In particular, it has been shown that T cells recognizing multiple regions of the spike, M and N protein of SARS-CoV-2 develop during COVID-19 (26, 33, 34) and that recognition of multiple epitopes is associated with milder symptoms (35). SARS-CoV-2-induced T cell immunity is maintained for at least six months and symptomatic primary infection is associated with higher levels of the persisting T cell response (36).

The lung and the gut are considered the main portals of entry for SARS-CoV-2 (37, 38). However, pulmonary manifestation dominates the clinical disease phenotype and only a subset of hospitalized patients suffers from gastrointestinal symptoms (2, 4). It is currently unclear, how infection of the lung alveolar and the intestinal epithelium contribute to the generation of the systemic immune response against SARS-CoV-2. Importantly, memory T and B cells primed in the gut are characterized by the expression of the gut-homing marker α4β7 integrin (20, 23). We therefore decided to study circulating T and B cell responses in hospitalized patients with COVID-19 to determine the contribution of SARS-CoV-2-associated intestinal lymphocyte imprinting based on gut-homing phenotypes.

In conclusion, our data show that adaptive immune cells expressing the gut homing integrin α4β7 are reduced in the peripheral blood of patients with COVID-19. It is very likely that α4β7 expression on memory lymphocytes is reflecting priming following antigen contact in the GALT, where the exclusive production of retinoic acid by DCs induces α4β7 integrin expression and imprints a gut homing phenotype. Thus, our data suggest that cells having received intestinal antigenic cues in the GALT are reduced in the peripheral blood during COVID-19. It is essential to keep in mind that this does not indicate that the cells have been in the gut tissue itself or that they will later home to the gut tissue.

Two main interpretations for this observation are conceivable: (1) α4β7-expressing lymphocytes are preferentially recruited from the circulation to peripheral tissues or (2) predominant (re-)circulation of lymphocytes not expressing α4β7 leads to a “dilution” of gut-imprinted cells.

The former option goes along with the clinical hallmark of lymphopenia in COVID-19, which is thought to arise from lymphocyte recruitment to tissues (31). Since CD4^+^ and CD8^+^ T and B cell frequencies expressing α4β7 decreased regardless of the presence of diarrhea as a symptom indicative of gastrointestinal involvement and fecal detection of virus RNA (39, 40), it is unlikely that such recruitment happens to the gut (30). However, it can still not be ruled out that α4β7^+^ cells might co-express other homing markers leading to their α4β7-independent uptake in other tissues or that they are eliminated from the blood by other mechanisms under inflammatory conditions. In this case, α4β7 expression might mark a lymphocyte population with particular pathogenetic relevance.

The second explanation would suggest that adaptive immune cells primed in the gut do at least numerically not play a major role for the systemic immune response against SARS-CoV-2 in COVID-19 even in patients, in which gastrointestinal involvement is likely. This would also be in line with the finding that IgA-expressing B cells with a gut homing phenotype did not contribute to the overall increase in IgA-expressing B cells observed in COVID-19 patients. One possible explanation might be that intestinal SARS-CoV-2 infection leads to the predominant generation of local immunity characterized by tissue-resident memory T cells (41) and, interestingly, a large part of intestinal T cells have a resident phenotype (42).

If this second interpretation is true, our findings might be interesting in the context of the ongoing efforts to develop efficient vaccines against SARS-CoV-2. While most candidate vaccines are designed as injections, some oral vaccines are also under development (e.g., VXA-CoV2-1). It has previously been shown for adenovirus subtypes and influenza that oral vaccines can also protect from pulmonary infections (43–45). Moreover, mucosal administration has been proposed to be more effective in inducing protective mucosal immunity (including the lung) than systemic application (46). It would then have to be doubted that this is similarly the case in COVID-19 in view of reduced gut-imprinted immunity in the circulation during active infection with the virus.

A limitation of our study is that most of our experiments did not assess SARS-CoV-2-specific T cells. However, our observations following virus-specific stimulation suggest that there is a clear correlation of α4β7 expression on overall memory CD4^+^ T cells with expression on SARS-CoV-2-specific memory CD4^+^ T cells. Moreover, the consistent alterations we observed during active infection and recovery in different patients as well as in a second patient cohort, strongly suggest that the effects are COVID-19-associated. It should also be noted that a certain difference in the age of our control group compared with the COVID-19 patients exists and age-related effects on integrin expression cannot be completely ruled out.

While lymphopenia is a hallmark of COVID-19 and has repeatedly been reported, lymphocyte subset analyses in the peripheral blood of patients with COVID-19 have only rarely been performed. In a cohort of 44 patients, Qin et al. reported reduced memory T cell levels in patients with severe compared to mild COVID-19, which were, however, still in the normal range (47). Similarly, Sekine et al. also observed decreased frequencies of memory T cells in patients with COVID-19, more in severe than in mild disease, and described the upregulation of markers such as CD38, CD69 or PD-1 (48). However, data on the expression of gut-homing markers in the context of SARS-CoV-2 infection have so far been missing.

Taken together, our findings suggest that gut-imprinted adaptive immune cells are eliminated from the circulation during COVID-19 hinting at a potential central role in its pathogenesis or that – in line with clinical symptoms – not the intestinal immune system, but other sites such as the lung dominate the shaping of systemic immune responses to SARS-CoV-2.

## Data Availability Statement

The raw data supporting the conclusions of this article will be made available by the authors, without undue reservation.

## Ethics Statement

The studies involving human participants were reviewed and approved by Ethics Committee of the Friedrich-Alexander University Erlangen-Nuremberg (174_20B). The patients/participants provided their written informed consent to participate in this study.

## Author Contributions

TM performed the experiments and analyzed the clinical data. TM, MN, and SZ designed the research. TM, EB, MW, LS, CV, SV, AK, MN, and SZ contributed samples or protocols and analyzed and interpreted the data. TM and SZ drafted the manuscript. All authors contributed to the article and approved the submitted version.

## Funding

German Research Foundation (DFG, ZU 377/4-1 to SZ, TRR241 TP C04 to CV and MFN), Interdisciplinary Center for Clinical Research (IZKF) of the University Erlangen-Nuremberg (J63, A84 to SZ), Wilhelm Sander-Stiftung (2020.045.1 to SV), “Bayerische Forschungsförderung” (SV), Bavarian State Ministry for Sciences and Art (TP-10 and TP-11 to AK), and National research network for University Medicine (NUM to AK).

## Conflict of Interest

The authors declare that the research was conducted in the absence of any commercial or financial relationships that could be constructed as a potential conflict of interest.

## Supplementary Material

Supplementary Figure 1 Lymphocyte subsets in PBMCs from COVID-19 and recovered patients compared to healthy controls. Representative (left) and quantitative (right) flow cytometry of the frequency of CD3^+^ **(A)** and CD3^+^CD4^+^ **(B)** T cells among lymphoid cells selected based on forward and sideward scatter, CD45RO^+^ cells among CD3^+^CD4^+^ T cells **(C)** and CD3^-^CD19^+^ B cells **(D)** among lymphoid cells. Each symbol represents an individual subject, n = 18 – 80 per group.

Supplementary Figure 2 Quantitative flow cytometry of the expression of the indicated markers stratified according to clinical parameters of COVID-19 patients. **(A)** Frequency of α4^+^β7^+^ and GPR15^+^ cells among CD3^+^CD4^+^ T cells according to oxygen saturation. **(B)** Frequency of α4β7 integrin-expressing CD3^+^CD4^+^CD45RO^+^ memory T cells according to respiratory rate and heart rate. **(C)** Frequency of IgA-expressing CD3^-^CD19^+^ B cells according to respiratory rate and heart rate. Each symbol represents an individual subject, n = 8 - 18 per group.

Supplementary Figure 3 Lymphocyte subsets in whole blood samples from patients with COVID-19 and healthy controls. Quantitative flow cytometry of the frequency (left) and absolute cell numbers (right) of CD3^+^ **(A)**, CD3^+^CD4^+^ **(B)** T cells among lymphoid cells selected based on forward and sideward scatter, CD45RO^+^ cells among CD3^+^CD4^+^ T cells **(C)** and CD3^-^CD19^+^ B cells **(D)** among lymphoid cells. Each symbol represents an individual subject, n = 10 – 31 per group.

Supplementary Figure 4 Lymphocyte subsets in whole blood samples from patients with COVID-19 and healthy controls. Representative (left) and quantitative (right) flow cytometry of the frequency and absolute cell numbers of CD3^+^CD8^+^ T cells among lymphoid cells selected based on forward and sideward scatter. Each symbol represents an individual subject, n = 10 – 31 per group.

## Abbreviations

CD, cluster of differentiation; COVID-19, coronavirus disease 2019; DCs, dendritic cells; GALT, gut-associated lymphoid tissue; MAdCAM-1, mucosal addressin vascular cell adhesion molecule 1; PD-1, programmed cell death protein 1; PBMCs, peripheral blood mononuclear cells; RALDH, retinaldehyde dehydrogenase; SARS-CoV-2, severe acute respiratory syndrome coronavirus 2; SEM, standard error of the mean; VDZ, vedolizumab.
