# Supplementary figures and images for: Circulating Adaptive Immune Cells Expressing the Gut Homing Marker α4β7 Integrin Are Decreased in COVID-19
Source: Front Immunol. 2021 Apr 20;12:639329. doi: 10.3389/fimmu.2021.639329 (PMC8093414; doi:10.3389/fimmu.2021.639329)

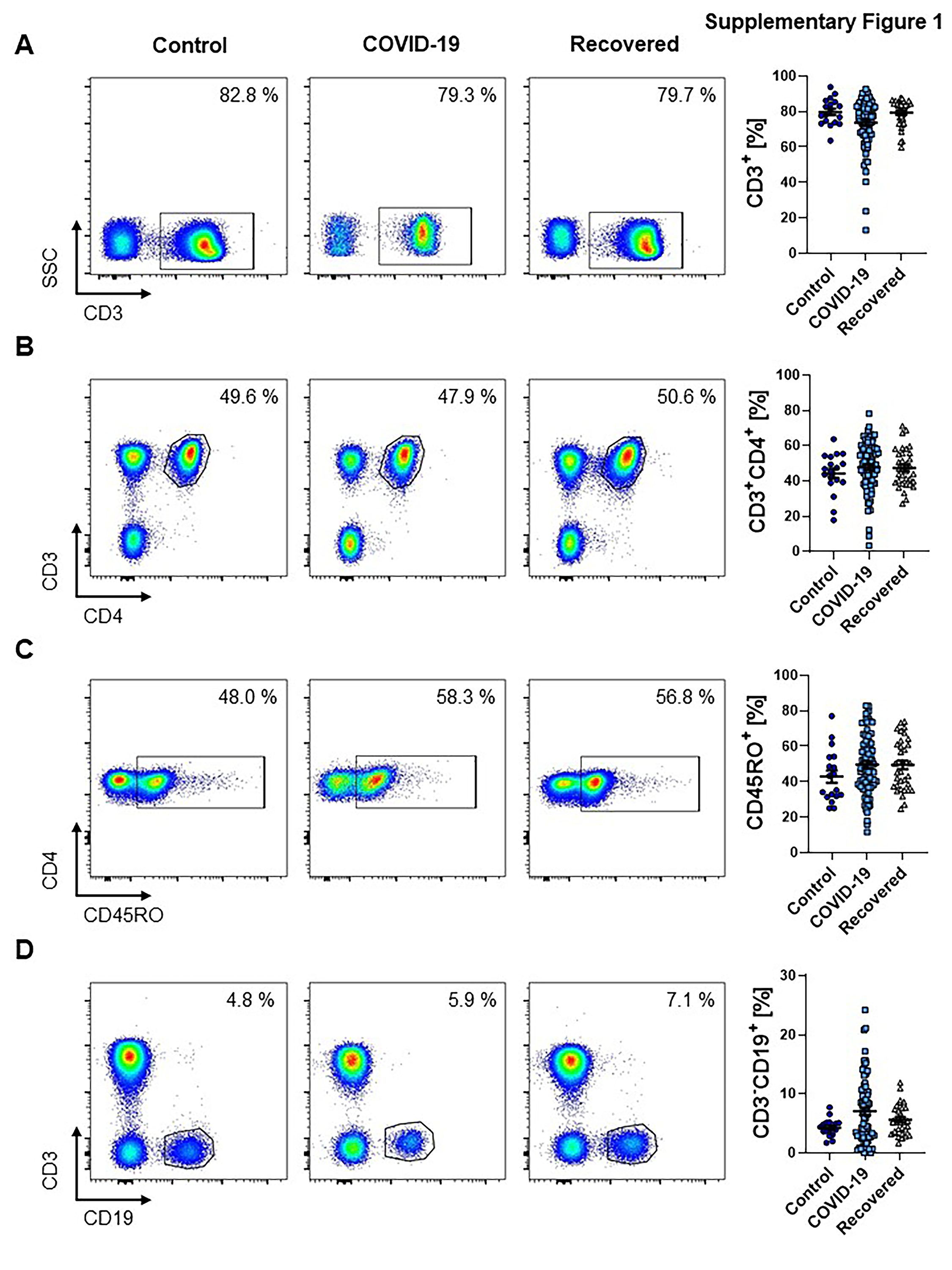

Supplement: Supplementary Figure 1 — Lymphocyte subsets in PBMCs from COVID-19 and recovered patients compared to healthy controls. Representative (left) and quantitative (right) flow cytometry of the frequency of CD3+ (A) and CD3+CD4+ (B) T cells among lymphoid cells selected based on forward and sideward scatter, CD45RO+ cells among CD3+CD4+ T cells (C) and CD3-CD19+ B cells (D) among lymphoid cells. Each symbol represents an individual subject, n = 18 – 80 per group. [file Image_1.tif]

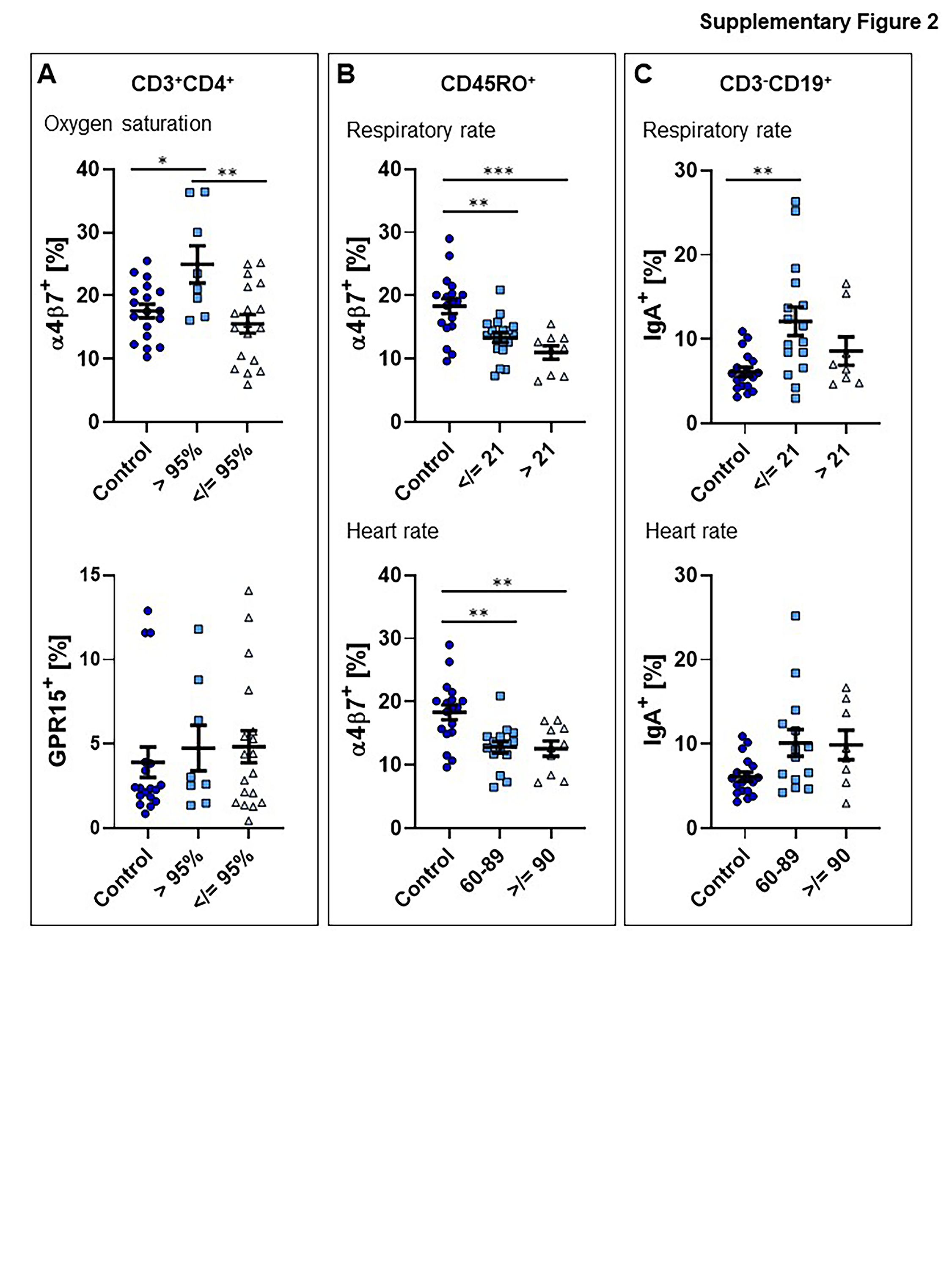

Supplement: Supplementary Figure 2 — Quantitative flow cytometry of the expression of the indicated markers stratified according to clinical parameters of COVID-19 patients. (A) Frequency of α4+β7+ and GPR15+ cells among CD3+CD4+ T cells according to oxygen saturation. (B) Frequency of α4β7 integrin-expressing CD3+CD4+CD45RO+ memory T cells according to respiratory rate and heart rate. (C) Frequency of IgA-expressing CD3-CD19+ B cells according to respiratory rate and heart rate. Each symbol represents an individual subject, n = 8 - 18 per group. [file Image_2.tif]

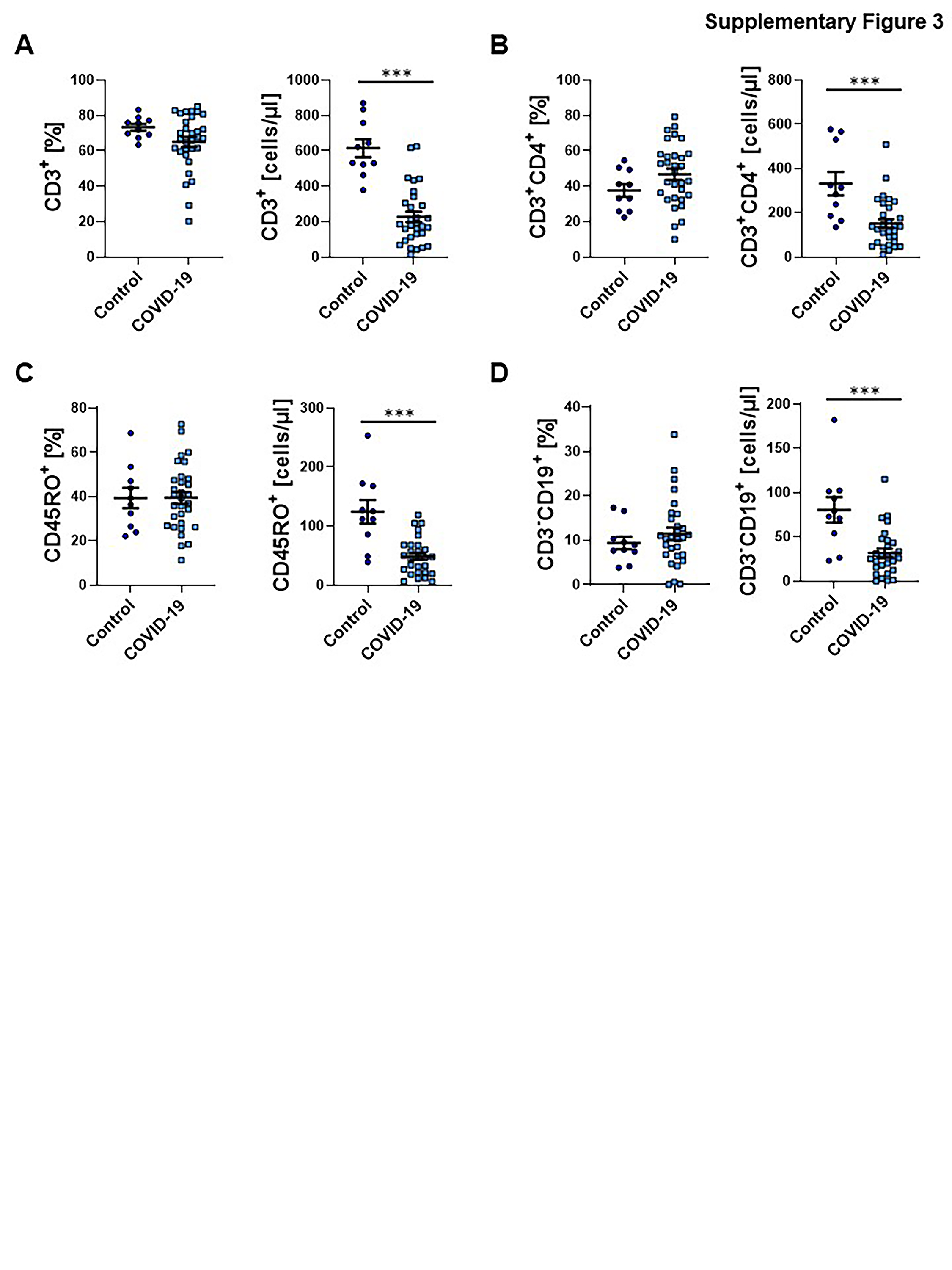

Supplement: Supplementary Figure 3 — Lymphocyte subsets in whole blood samples from patients with COVID-19 and healthy controls. Quantitative flow cytometry of the frequency (left) and absolute cell numbers (right) of CD3+ (A), CD3+CD4+ (B) T cells among lymphoid cells selected based on forward and sideward scatter, CD45RO+ cells among CD3+CD4+ T cells (C) and CD3-CD19+ B cells (D) among lymphoid cells. Each symbol represents an individual subject, n = 10 – 31 per group. [file Image_3.tif]

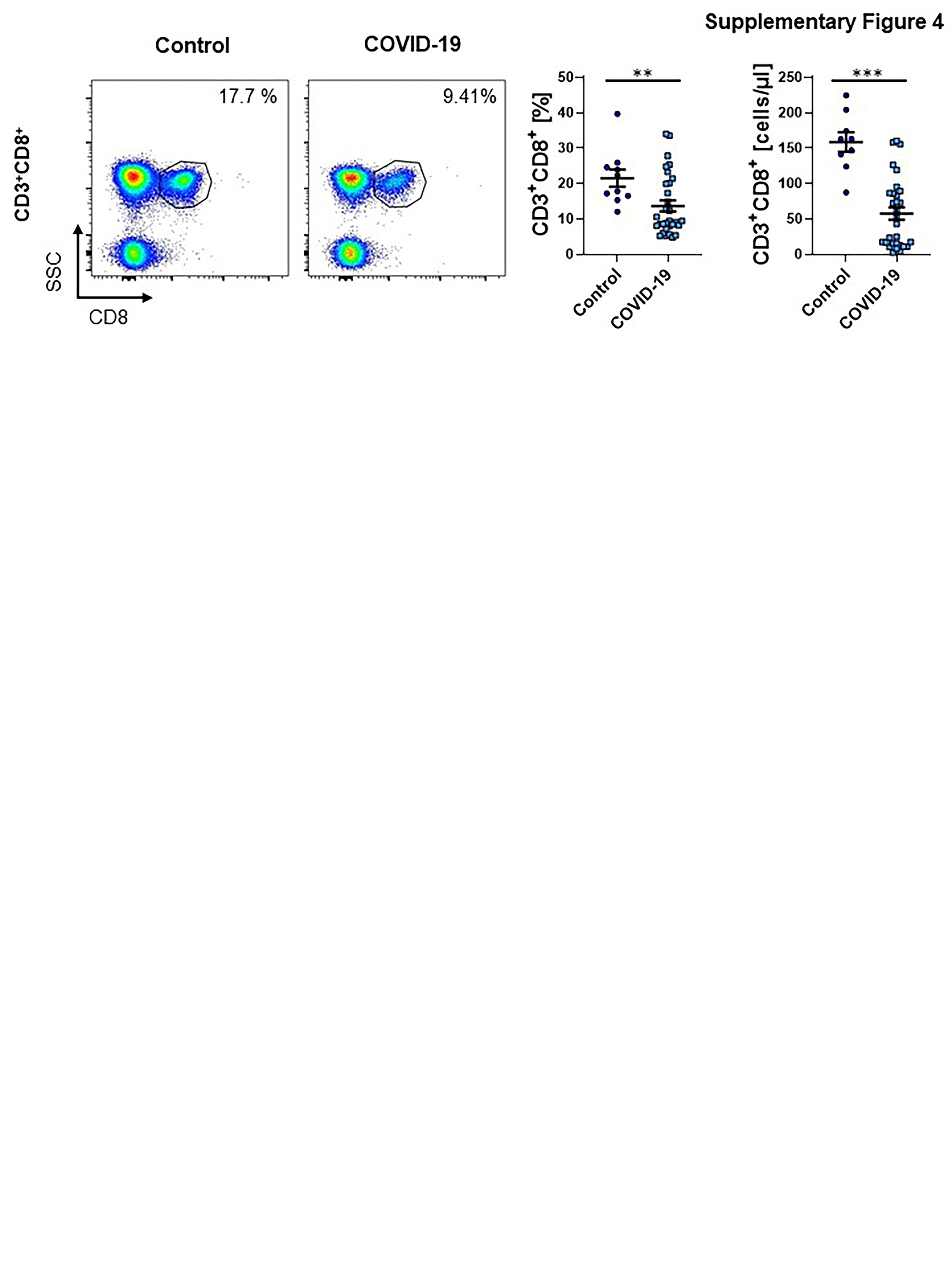

Supplement: Supplementary Figure 4 — Lymphocyte subsets in whole blood samples from patients with COVID-19 and healthy controls. Representative (left) and quantitative (right) flow cytometry of the frequency and absolute cell numbers of CD3+CD8+ T cells among lymphoid cells selected based on forward and sideward scatter. Each symbol represents an individual subject, n = 10 – 31 per group. [file Image_4.tif]
